# Sevoflurane modulates breast cancer cell survival via modulation of intracellular calcium homeostasis

**DOI:** 10.1186/s12871-020-01139-y

**Published:** 2020-09-29

**Authors:** Xiaoqian Deng, Megha Vipani, Ge Liang, Divakara Gouda, Beibei Wang, Huafeng Wei

**Affiliations:** 1 Department of Anesthesiology and Critical Care, Perelman School of Medicine, University of Pennsylvania, 305 John Morgan Building, 3610 Hamilton Walk, Philadelphia, PA 19104 USA; 2 Department of Anesthesiology, West China Hospital of Sichuan University, Chengdu, Sichuan China; 3 University of Virginia School of Medicine, Charlottesville, VA 22903 USA; 4 Department of Obstetrics and Gynecology, Tongji Hospital, Huazhong Science and Technology University, Wuhan, China

**Keywords:** Anesthetics, Breast, Cancer, Survival, Metastasis, Ca^2 +^, TRP Channel

## Abstract

**Background:**

Some retrospective and in vitro studies suggest that general anesthetics influence breast cancer recurrence and metastasis. We compared the effects of general anesthetics sevoflurane versus propofol on breast cancer cell survival, proliferation and invasion in vitro. The investigation focused on effects in intracellular Ca^2+^ homeostasis as a mechanism for general anesthetic-mediated effects on breast cancer cell survival and metastasis.

**Methods:**

Estrogen receptor-positive (MCF7) and estrogen receptor-negative (MDA-MB-436) human breast cancer cell lines along with normal breast tissue (MCF10A) were used. Cells were exposed to sevoflurane or propofol at clinically relevant and extreme doses and durations for dose- and time-dependence studies. Cell survival, proliferation and migration following anesthetic exposure were assessed. Intracellular and extracellular Ca^2+^ concentrations were modulated using Ca^2+^ chelation and a TRPV1 Ca^2+^ channel antagonist to examine the role of Ca^2+^ in mediating anesthetic effects.

**Results:**

Sevoflurane affected breast cancer cell survival in dose-, time- and cell type-dependent manners. Sevoflurane, but not propofol, at equipotent and clinically relevant doses (2% vs. 2 μM) for 6 h significantly promoted breast cell survival in all three types of cells. Paradoxically, extreme exposure to sevoflurane (4%, 24 h) decreased survival in all three cell lines. Chelation of cytosolic Ca^2+^ dramatically decreased cell survival in both breast cancer lines but not control cells. Inhibition of TRPV1 receptors significantly reduced cell survival in all cell types, an effect that was partially reversed by equipotent sevoflurane but not propofol. Six-hour exposure to sevoflurane or propofol did not affect cell proliferation, metastasis or TRPV1 protein expression in any type of cell.

**Conclusion:**

Sevoflurane, but not propofol, at clinically relevant concentrations and durations, increased survival of breast cancer cells in vitro but had no effect on cell proliferation, migration or TRPV1 expression. Breast cancer cells require higher cytoplasmic Ca^2+^ levels for survival than normal breast tissue. Sevoflurane affects breast cancer cell survival via modulation of intracellular Ca^2+^ homeostasis.

## Background

Breast cancer is the most common malignancy in women worldwide and a leading cause of cancer-related death in this population. Surgical resection is the primary and curative treatment for patients with localized disease. Multiple factors in the perioperative period including the use of volatile anesthetics and opioids can suppress immune function and facilitate tumor growth [1–4]. Regional analgesics attenuate the surgical stress response leading to hypotheses that they may be favorable over alternative anesthetics [5–7]. In vitro models demonstrate enhanced breast cancer cell function with exposure to an inhalational anesthetic [8]. Serum from patients receiving regional analgesia and propofol inhibited breast cancer cell proliferation to a greater extent than that of patients receiving volatile anesthetics [9]. Retrospective analyses demonstrate the reduced 5-year risk of recurrence of breast cancer with the use of propofol-based total intravenous anesthesia when compared with sevoflurane anesthesia for modified radical mastectomy [10]. Overall, retrospective and cohort clinical studies comparing anesthetic techniques for breast cancer lack concurrence on the benefits of intravenous propofol over inhalational anesthetics [1, 3–5, 7, 11, 12]. These findings call for further investigation into the mechanisms through which anesthetic techniques may alter outcomes for patients with breast cancer.

We focused our study on Ca^2+^ homeostasis as a mechanism for the action of general anesthetics on breast cancer cell functions. Ca^2+^ ions are a key mediator of numerous cellular processes including proliferation and apoptosis [13]. Changes in complex Ca^2+^ signaling pathways and Ca^2+^ transport proteins contribute to breast tumorigenesis [14, 15]. Transient receptor potential (TRP) channels are a family of ion channels that have been implicated in oncogenic conversion of breast cells [15, 16]. Specifically, transient receptor potential vanilloid 1 (TRPV1) is a Ca^2+^ transport protein expressed in high levels in various aggressive breast cancer cell lines compared to normal breast epithelial cells [15]. Therapies targeting cellular Ca^2+^ pathways have demonstrated anti-tumor efficacy in vitro*.* Volatile anesthetics, such as sevoflurane, isoflurane, and desflurane, and propofol activate Ca^2+^ receptors [17, 18] including TRPV1, which is sensitized by exposure to inhaled anesthetics [19]. We hypothesized that the highly selective plasma membrane Ca^2+^ channel, TRPV1, may mediate the observed effects of general anesthetics.

We compared the effects of exposure to two commonly used general anesthetics, sevoflurane and propofol, on breast cancer cell survival, proliferation and migration at equipotent and clinically relevant concentrations in vitro and investigated the role of intracellular Ca^2+^ homeostasis and TRPV1 Ca^2+^ channels.

## Methods

### Reagents

Culture media and BAPTA-AM was obtained from Life Technologies (Carlsbad, CA, USA). Propofol was obtained from Sigma (St. Louis, MO, USA). Sevoflurane was obtained from Midwest Veterinary Supply Inc. (Norristown, PA, USA). MTT (Sigma-Aldrich, St. Louis, MO, USA), DAPI (Sigma, St. Louis, MO, USA) and BrdU (Invitrogen, Carlsbad, CA, USA) assays were used to evaluate breast cancer cells following anesthetic exposure. SB-366791 was obtained from Tocris Bioscience (Minneapolis, MN, USA).

### Cell culture

An estrogen receptor-positive human breast adenocarcinoma cell line, MCF7, and an estrogen receptor-negative human breast adenocarcinoma cell line, MDA-MB-436, were used in this study. A normal human breast epithelial line, MCF10A, was used as control. Cell lines were obtained from American Type Culture Collection (Gaithersburg, Maryland, USA). MCF10A cells were cultured in DMEM/F12 Nutrient Mixture supplemented with 5% Horse Serum, EGF 20 ng/ml, insulin 10 μg/ml, hydrocortisone 0.5 mg/ml, cholera toxin 100 ng/ml, MCF7 cells were cultured in MEM with 10% fetal bovine serum (FBS) and 10μg/ml insulin. MDA-MB-436 cells were cultured in DMEM with 10% FBS. The mediums were supplemented with 1% penicillin/streptomycin. Cells were maintained in a humidified incubator at 37 °C in 95% air and 5% CO_2_. The medium was changed every two days and the maximum cell passage was 15. Cells were harvested and added to assay plates before experimentation.

### Anesthetic exposure

The three cell lines were exposed to either sevoflurane or propofol in a gas-tight chamber stored in a gastight chamber inside the culture incubator (Bellco Glass, Inc., Vineland, NJ), with humidified 5% CO_2_–21% O_2_-balanced N_2_ (AirGas East, Bellmawr, NJ) going through a calibrated agent-specific vaporizer. All three cell-lines were exposed to either 1, 2% or 4% sevoflurane or 1, 2, and 4 μM propofol for dose-dependent studies. Exposures lasted for 3, 6, and 24 h at each anesthetic concentration for time-dependent studies. Gas-phase concentrations in the gas chamber were verified and maintained at the desired concentration throughout the experiments using an infrared Ohmeda 5330 agent monitor (Coast to Coast Medical, Fall River, MA). Concentrations of 2% sevoflurane and 2 μM propofol and a duration of 6 h were selected for subsequent studies as they represented equipotent clinically relevant exposures [4, 6, 7].

### Determination of cell viability

The 3-(4,5-dimethylthiazol-2yl)-2,5-diphenyltetrazolium bromide (MTT) colorimetric assay was used to assess for cell viability. MTT (0.5 mg/mL) was added to the growth medium in 24-well plates containing either treated or control cells. Following incubation for 3 h at 37 °C, formazan crystals were dissolved in dimethyl sulfoxide (DMSO). Optical density was measured at 590 nm using a SynergyTM H1 microplate reader (BioTek) [8, 9]. The data are presented as percentage of control.

### Determination of cell proliferation

Cell proliferation was determined with the 5-bromo-2’deoxyuridine (BrdU) immunostaining assay [8, 9]. Cells were plated onto slides in the medium for 24 h after treatment. BrdU labeling solution (10 μM) was added to the medium and cells were incubated for 3 h. Cells were then fixed with 4% paraformaldehyde in PBS and permeabilized using 0.1% Triton X-100 in PBS. The cells were incubated overnight with rat monoclonal anti-5-bromodeoxyuridine primary antibody (1:100) (Santa Cruz Biotechnology, Dallas, TX, USA) at 4 °C. The cells were washed with 0.1% Triton X-100 in PBS, and BrdU was detected with fluorescently labeled secondary antibody conjugated with anti-rat IgG (1:1000 for 1 h). The immunostained cells were mounted on microscope slides with Prolong Gold Antifade Reagent containing 4′,6-diamidino-2-phenylindole (DAPI) for visualization of cell nuclei. The imaging of cells were taken using an Olympus BX41TF fluorescence microscope (400×; Olympus, Waltham, Massachusetts, USA) equipped with iVision software (Biovision Technologies, Exton, PA, USA). The number of DAPI-labeled cells and the number of 5-bromodeoxyuridine-labeled cells were counted, and the mean number of cells was calculated from 5 random areas of each coverslip, with 3 to 4 coverslips per condition, from 3 to 4 different cultures. The experimental n equals the number of coverslips. The data are expressed as the percentages of the number of 5-bromodeoxyuridine-positive cells to the total number of cells.

### Determination of cell migration

The Transwell Migration Assay was used to determine the invasiveness of cell lines following treatment. A transwell chamber with polycarbonate membrane filters was used for the assay. Cells suspended in serum-free medium were added to the upper chamber and cells suspended in medium containing 10% FBS was added to the lower chamber. A cotton swab was used to remove the cells remained on the upper face of the filters of the membrane after 24 h. The cells that penetrated across the polycarbonate membrane were fixed with 4% formaldehyde for 15 min and stained with 0.1% crystal violet for 20 min. Five random fields were selected; invaded cells were counted under an Olympus BX41TF fluorescence microscope (100x) equipped with iVision software. The experiments were repeated at least three times.

### Calcium modulation

In subsequent experiments, we tested the hypothesis that the effects of the general anesthetics are mediated by intracellular Ca^2+^ signaling. Cells were pre-treated with 5 μM BAPTA-AM Ca^2+^ chelator for 30 min before exposure to a general anesthetic. To determine the role of extracellular Ca^2+^ in the effects of general anesthetics, assays were performed using cells incubated in Ca^2+^ free medium. Cells were pretreated with 10 μM SB-366791, a TRPV1 Ca^2+^ channel selective antagonist, for 1 h before anesthetic exposure to evaluate the contribution of the channel on cancer cell functions [12, 13].

### Western blot

The expression of TRPV1 was evaluated using Western blot analysis. Following anesthetic treatment, the cell culture plate on ice was washed once with ice-cold PBS. After aspiration of PBS, 200 μl of ice-cold lysis buffer (50 mM Tris/HCl, pH 7.8, 150 mM NaCl, 1% Triton X-100) was added to each well and maintained on ice for 5 min. The homogenate was collected with a cell scraper and then centrifuged at 4 °C in a microcentrifuge at 12,000 g for 30 min. The supernatant was gently collected and preserved at − 70 °C for future use. Protein concentration was determined with a bicinchoninic acid kit (Thermo Fisher Scientific, Waltham, MA, USA). For electrophoresis, 40 μg of protein from different samples were loaded on 15% sodium dodecyl sulfate-polyacrylamide gel electrophoresis gels and run with a constant current, and then the protein was transferred to nitrocellulose membranes (Bio-Rad, Hercules, CA, USA) using a wet transfer system (Bio-Rad). The membranes were blocked with 5% nonfat dry milk dissolved in PBS with 0.2% TWEEN 20 for 1 h at room temperature and incubated overnight at 4 °C with the primary rabbit monoclonal antibody against TRPV1 (1:1000) (Cell Signaling Technology, Danvers, MA, USA) or primary mouse monoclonal antibody against β-actin (1:3000) (Santa Cruz Biotechnology, Dallas, TX, USA). This was followed by a wash with secondary antibody conjugated with anti-rabbit and anti-mouse IgG conjugated with horseradish peroxidase (1:10,000) (Bio-Rad) at room temperature for 1 h. The protein on the membranes was detected in a Kodak Image Station 4000MM Pro (Kodak, USA) ECL Prime Western blotting detection reagent (GE Healthcare, UK), and images were acquired with Carestream imaging software (Carestream Health, USA). Signal intensity was quantitatively analyzed with ImageJ software, and the β-actin loading control was used for normalization. n equals the number of wells for each condition.

### Statistical analysis

All data were expressed as means ± standard deviation from a minimum of three separate experiments. Statistical analysis was performed using Graphpad Prism 6. Results with *p* < 0.05 were considered statistically significant.

## Results

### Cell viability

**Fig. 1:**
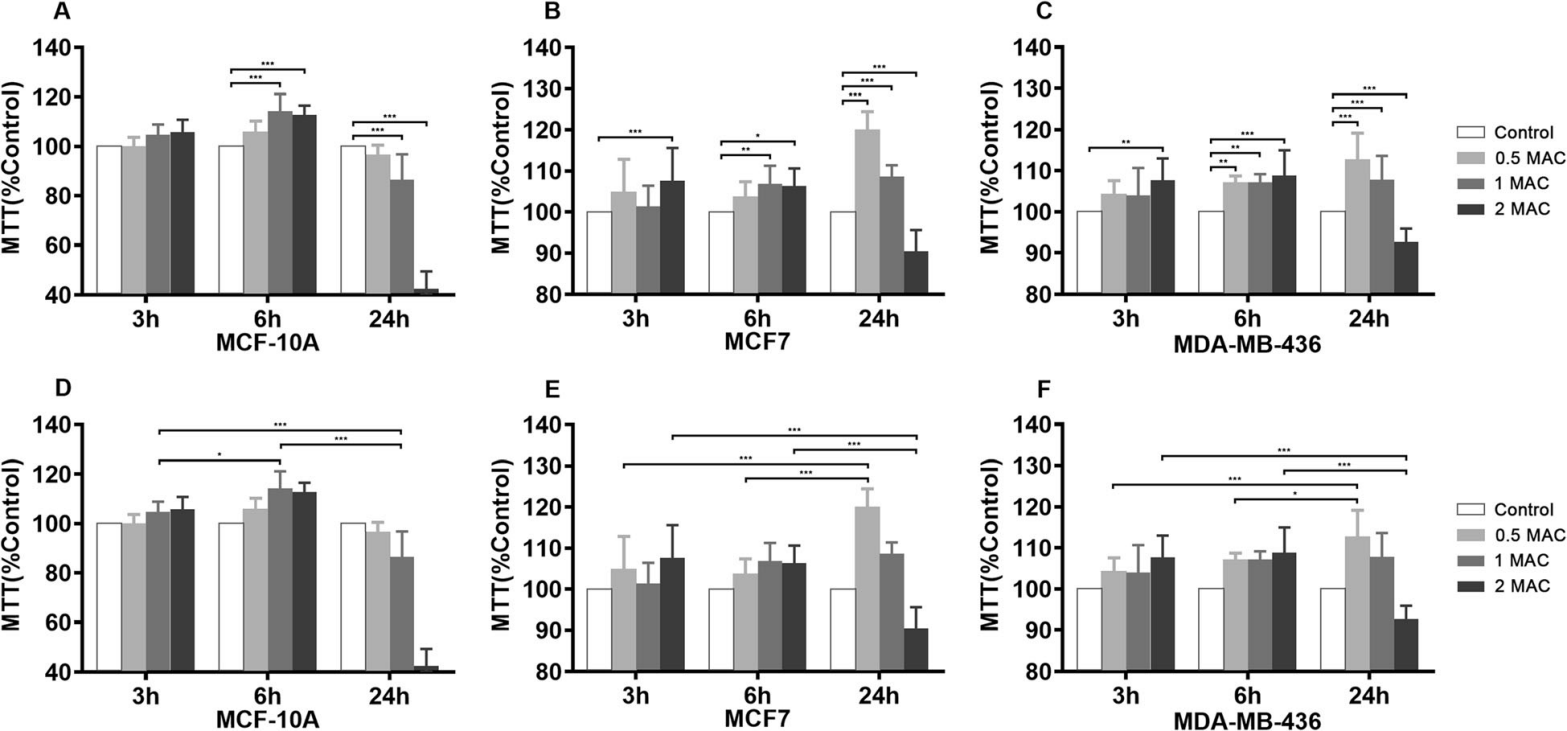
Sevoflurane modulates breast cancer cell survival in a dose- and time-dependent manner. **a**, **b**, **c** Dose-dependence relationships of sevoflurane exposure on breast cancer survival in vitro. **d**, **e**, **f** Time-dependence relationships of sevoflurane exposure on breast cancer cell survival in vitro. Exposure to sevoflurane at clinically relevant concentrations of 2 and 4% for 6 h significantly promoted breast cancer survival in normal breast cells (MCF10A) along with estrogen receptor-positive (MCF7) and estrogen receptor-negative (MDA-MB-436) breast adenocarcinoma cells. Treatment with 4% sevoflurane produced a statistically significant increase in cell survival after 3 h in both breast cancer cell types. Exposure to low-dose sevoflurane (1%) for an extended duration of 24 h also increased survival when compared to treatment for 3 and 6 h for the breast adenocarcinoma lines. Paradoxically, extreme exposure to 4% sevoflurane for 24 h reduced cell survival in all cell lines. All data are expressed as means ± SD from at least three separate experiments in duplicate or triplicate and analyzed by two-way ANOVA followed by Tukey multiple comparison tests. * *P* < 0.5, ** *P* < 0.01, *** *P* < 0.001, **** *P* < 0.0001

**Fig. 2:**
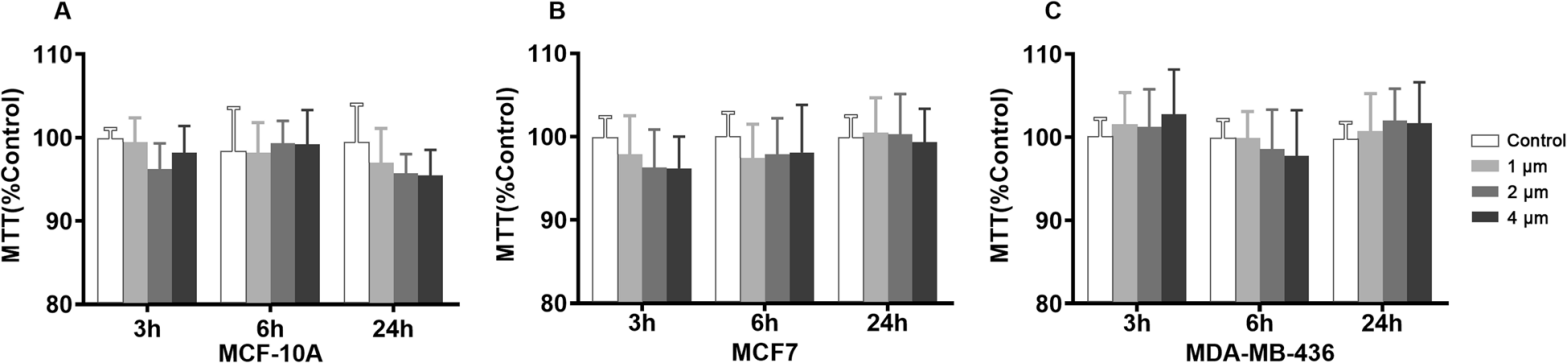
Exposure to propofol does not affect breast cancer cell survival. Dose- and time-dependence experiments with exposure to 1, 2, and 4 μM propofol for 3, 6, and 24 h demonstrated that propofol does not modulate cell survival in normal breast tissue (**a**) or breast adenocarcinoma lines (**b**, **c**)

Treatment with sevoflurane impacted breast cancer cell viability as determined by the MTT colorimetric assay in a dose-dependent (Fig. 1a, b, c) and time-dependent (Fig. 1d, e, f) manners. The normal breast tissue cell line (MCF10A) along with the estrogen receptor-positive (MCF7) and estrogen receptor-negative (MDA-MB-436) cell lines demonstrated increased viability following exposure to either 2% or 4% sevoflurane for 6 h when compared to the control group (MCF10A 1MAC&2MAC *P* < 0.0001, MCF7 1MAC *P* = 0.0002, 2MAC *P* = 0.0021, MDA-MB-436 1MAC *P* = 0.0002, 2MAC *P* < 0.0001). Sevoflurane treatment duration of 3 h produced a statistically significant effect at a concentration of 4% for the two breast cancer cell lines (MCF7 *P* < 0.0001, MDA-MB-436 *P* = 0.0004). A paradoxical reduction in cell viability was observed following exposure to 4% sevoflurane for a prolonged duration of 24 h in all three cell types (MCF10A&MCF7 P < 0.0001, MDA-MB-436 *P* = 0.0009). Treatment with propofol at equipotent and clinically relevant doses had no significant effect on breast cancer cell viability (Fig. 2).

### Calcium homeostasis and cell viability

**Fig. 3:**
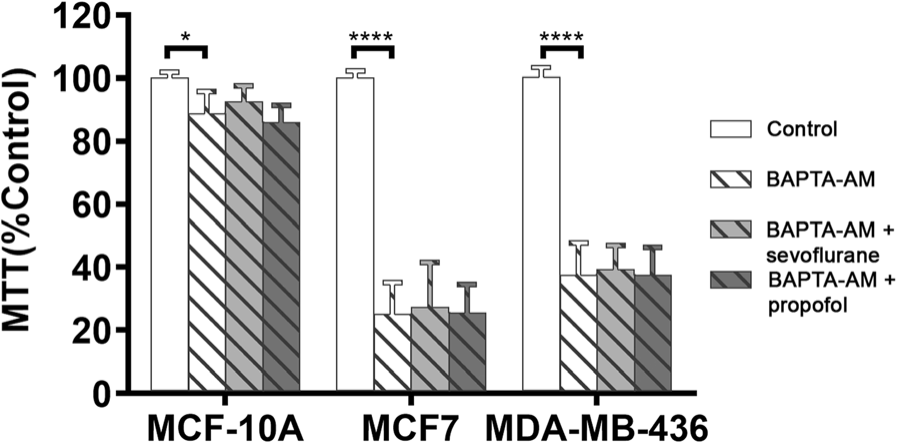
Decreased cytosolic Ca^2+^ concentrations reduce breast cancer cell survival. Chelation of cytosolic Ca^2+^ with BAPTA-AM dramatically reduced cell survival in both breast cancer lines but not in normal breast tissue. The use of general anesthetics sevoflurane and propofol did not alter this effect. Data represents means ± SD from at least 9 repeats of 3 separate experiments and was analyzed by two-way ANOVA followed by Turkey multiple comparison tests. * *P* < 0.05, **** P < 0.0001

**Fig. 4:**
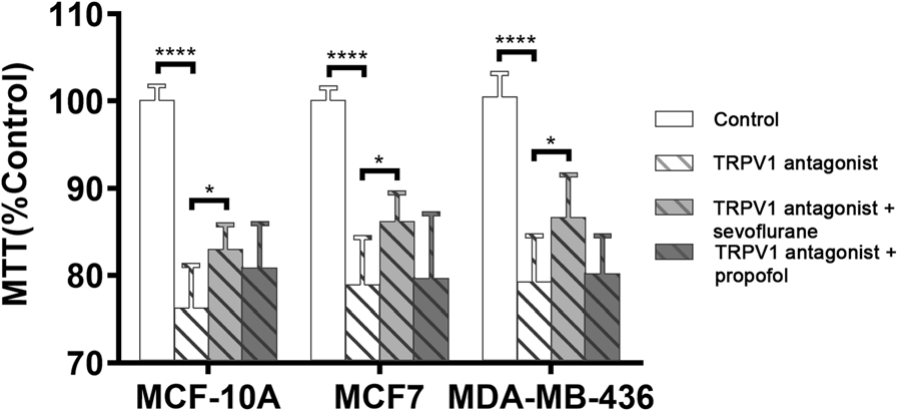
Sevoflurane partially reverses the reduction in cell survival by TRPV1 channel antagonism. Inhibition of Ca^2+^ influx through the TRPV1 receptor Ca^2+^ channel with the selective antagonist SB-366791 reduces cell survival in all three cell lines. Concomitant use of sevoflurane, but not propofol, partially reversed this effect. Data represents means ± SD from at least 9 repeats of 3 separate experiments and was analyzed by two-way ANOVA followed by Turkey multiple comparison tests. * P < 0.05, **** P < 0.0001

To evaluate the role of intracellular Ca^2+^ homeostasis in the augmentation of breast cancer cell viability by sevoflurane, we modulated intracellular and extracellular Ca^2+^ concentrations. For these experiments, 2% sevoflurane and 2 μM propofol were chosen as equipotent and clinically relevant anesthetic concentrations along with a treatment duration of 6 h. Increased cell viability was demonstrated following treatment with 2% sevoflurane for 6 h when compared to the control (MCF10A *p* < 0.0001, MCF7 & MDA-MB-436 *p* = 0.0002). Treatment with 2 μM propofol had no significant effect on cell viability. The addition of BAPTA-AM Ca^2+^ chelator dramatically reduced the viability of MCF7 and MDA-MB-436 breast cancer cell lines (MCF7 & MDA-MB-436 *p* < 0.0001) but did not impact that of the MCF10A normal breast tissue line (Fig. 3). To elucidate the mechanism of sevoflurane on breast cancer cell viability, we studied the role of cell membrane Ca^2+^ channel TRPV1 activation on cell survival. Similar to the prior Ca^2+^ modulation techniques, the highly specific TRPV1 channel antagonist SB-366791 reduced cell viability in all three cell lines (MCF10A, MCF 7 & MDA-MB-436 *p* < 0.0001). Concurrent treatment with sevoflurane and the TRPV1 channel antagonist partially reversed this reduction in cell survival (MCF 10A *p* = 0.022, MCF7 *p* = 0.0148, MDA-MB-436 *p* = 0.0128) (Fig. 4). The use of propofol with SB-366791 did not impact cancer cell viability when compared to the use of SB-366791 alone.

### Cell proliferation

**Fig. 5:**
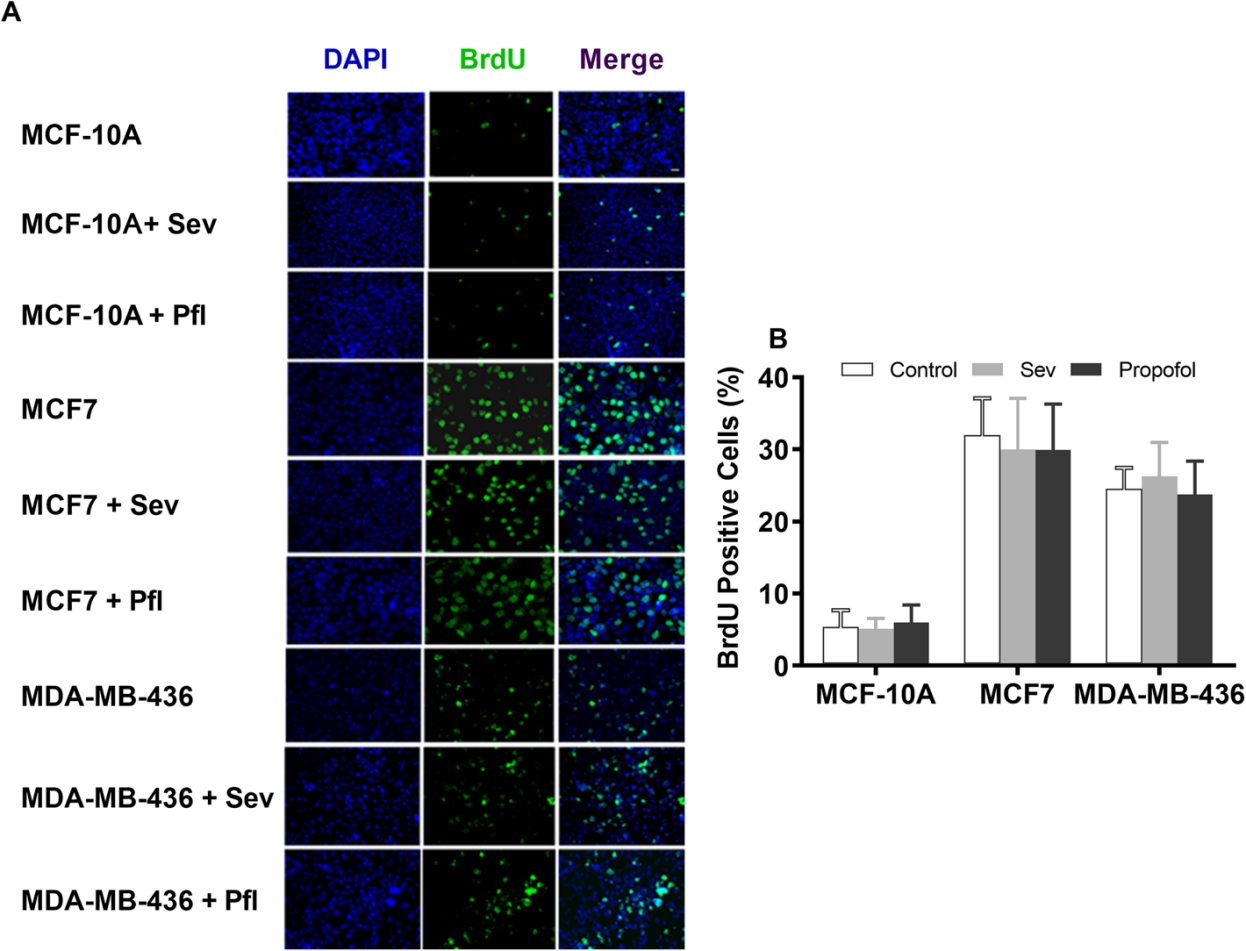
Effects of general anesthetics (GAs) on breast cancer cell proliferation. **a** Representative images showing the double immunostaining of cell nuclei with 4′,6-diamino-2-phenylindole (DAPI, blue, arrows) and 5-bromodeoxyuridine (BrdU, green, arrows) in control breast cancer cells (MCF 10A), estrogen receptor-positive (MCF 7) and negative (MDA-MB-436) breast cancer cells with and without sevoflurane (2%) and propofol (2 μM), at equipotent dose for 6 h. Scale bar = 100 μm. **b** Quantitative analysis of the effect of a 6-h exposure to an equipotent dose of sevoflurane (2%) vs. propofol (2 μM) on cell proliferation. Exposure to 2% sevoflurane and 2 μM propofol does not affect the percentage of cell proliferation (% of BrdU-positive cells) among all three cell lines (*n* = 12). All data are expressed as means ± SD from at least three separate experiments in duplicate or triplicate and analyzed by one-way ANOVA followed by Tukey multiple comparison tests

The effects of a 6-h exposure to equipotent doses of sevoflurane (2%) and propofol (2 μM) on breast cell proliferation were evaluated using immunostaining with diamino-2-phenylindole (DAPI, blue) and 5-bromodeoxyuridine (BrdU, green) (Fig. 5). Quantitative analysis of stained cells demonstrated no significant difference in the percentage of BrdU-positive cells between two general anesthetics among all three cell lines.

### Cell invasion

**Fig. 6:**
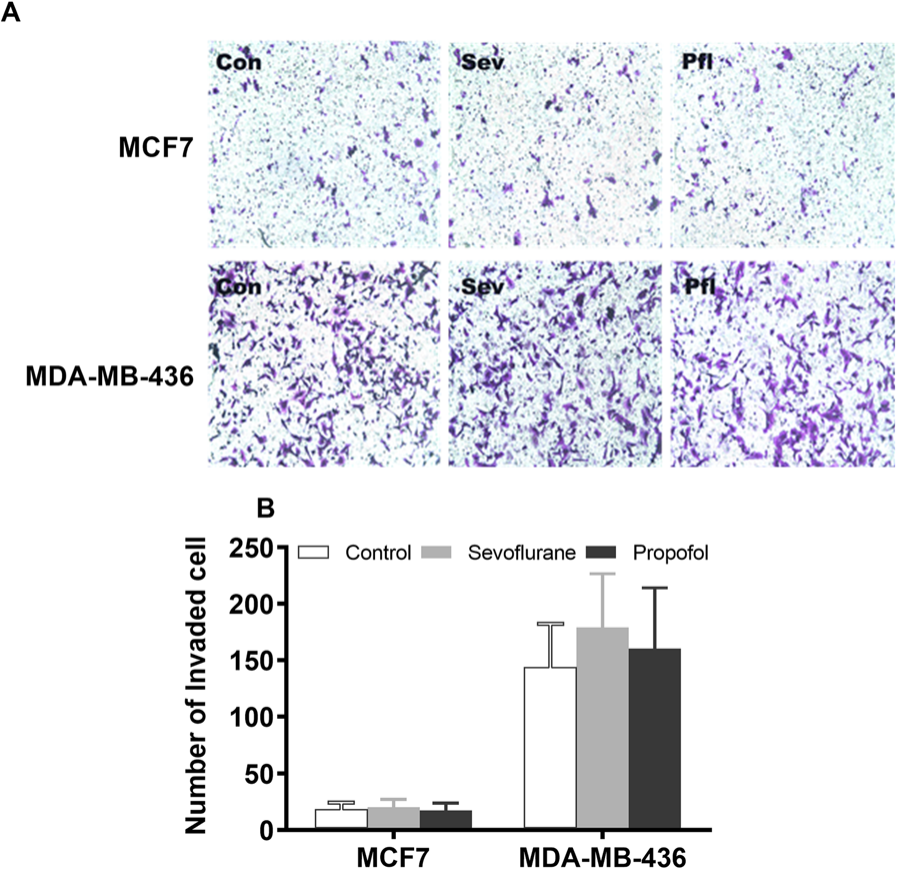
Effects of general anesthetics (GAs) on breast cancer cell migration. **a** Representative images of the Transwell Migration Assay used to determine the invasiveness of estrogen receptor-positive (MCF 7) and negative breast cancer cell lines (MDA-MB-436). **b** MDA-MB-436 exhibits greater migration potential thanMCF7. Exposure to equipotent sevoflurane (2%) and propofol (2 μM) did not affect migration in either breast cancer cells. All data are expressed as means ± SD from at least repeats of 3 separate experiments in duplicate or triplicate wells, and analyzed by one-way ANOVA followed by Tukey multiple comparison tests

The Transwell Migration Assay was used to determine the invasiveness of breast cancer cell lines following exposure to general anesthetics (Fig. 6). The estrogen receptor-negative MDA-MB-436 has a greater invasion potential that the estrogen receptor-positive MCF7. Treatment with equipotent doses of sevoflurane (2%) and propofol (2 μM) for 6 h did not affect the invasion capacity in either of two breast cancer cell lines.

### TRPV1 channel protein expression

**Fig. 7:**
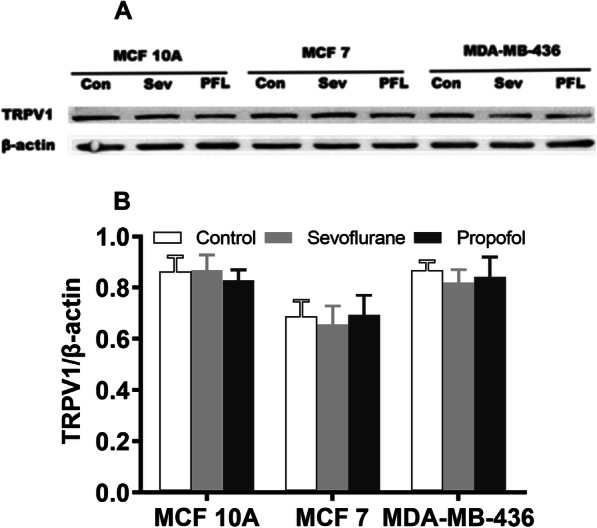
Effect of general anesthetics on TRPV1 channel expression. **a** TRPV1 protein expression in all three cell lines was quantified using the cropping of the Western blot. The β-actin was used as a loading control. Full-length blots are presented in Supplementary Fig. 8. **b** Treatment with equipotent doses of sevoflurane (2%) and propofol (2 μM) for 6 h did not affect TRPV1 protein expression in any of the three cell lines. All values shown are means ± SD of triplicate repeats from three separate experiments and were analyzed by one-way ANOVA

As the TRPV1 channel plays an important role in tumor cell survival, proliferation and migration, we examined the changes in TRPV1 protein levels. Expression of the cell membrane Ca^2+^ channel TRPV1 was quantified using Western blot (Fig. 7). Exposure to the general anesthetics (sevoflurane vs. propofol) did not significantly alter TRPV1 expression in the three cell lines.

## Discussion

Anesthetic technique may influence long-term recurrence and outcomes following surgical interventions for breast cancer. The emerging research on dysregulated Ca^2+^ signaling in breast oncogenesis along with anesthetic modulation of Ca^2+^ channels led to the hypothesis that Ca^2+^ homeostasis may be a potential mechanism. This study aimed to examine the effects of general anesthetics on breast cancer cell function and to characterize the role of Ca^2+^ homeostasis in this relationship. Sevoflurane increases breast cancer cell survival at clinically relevant concentrations and durations in both estrogen receptor-positive and negative breast cancer cells in vitro while propofol has no such effect. Exposure to sevoflurane at extreme concentrations and durations paradoxically decreases the survival of breast adenocarcinoma cells. Neither sevoflurane nor propofol has significant effects on proliferation, migration or TRPV1 protein expression in the studied cell lines.

In various kinds of neurons, general anesthetics at low concentrations for short durations promote cell survival, proliferation and neurogenesis by adequate or physiological increase of cytosolic Ca^2+^ concentrations via activation of InsP_3_ receptor (InsP_3_R) Ca^2+^ channel and subsequent Ca^2+^ release from the endoplasmic reticulum (ER) [20–23] or Ca^2+^ influx from extracellular space [24], thus resulting in preconditioning cytoprotection [25] or promoting autophagy [18]. However, general anesthetics at high concentrations for prolonged duration cause cell death directly by pathological Ca^2+^ release from the ER via excessive activation of InsP_3_R [17, 20] or abnormal Ca^2+^ influx from extracellular space. Similar to the dual effects of general anesthetics on cell survival in most neurons, the results from this study suggest that commonly used general anesthetic sevoflurane at low concentrations for short durations also promotes breast cancer survival, while at high concentrations for prolonged durations causes damage to these cells. This dual effect of cytoprotection versus cytotoxicity depends on exposure levels. Similar to the results of neuronal studies [21, 23, 24], the inhalational anesthetic sevoflurane was a more potent modifier of breast cancer cell survival in vitro than propofol. These results suggest general anesthetics have similar effects on cell survival in different cell types.

In various in vitro and in vivo models, sevoflurane has been shown to either promote [8, 26] or inhibit [27] breast cancer cell proliferation and/or metastasis. Compared to sevoflurane, propofol has been demonstrated to be less likely to promote breast cancer cell proliferation [26] and reduce the risk of recurrence during the initial 5 years after modified radical mastectomy [10]. The results from the present study suggest that propofol at clinically relevant concentrations and durations is less likely to promote breast cancer survival than sevoflurane, which is consistent with previous findings that propofol decreases the risk of proliferation and tumor recurrence [10, 26]. Our dose- and time-dependence experiments also revealed that exposure to sevoflurane at a high concentration for an extended period paradoxically and dramatically decreased cell survival. Prior physiologic studies demonstrate that prolonged rises in intracellular Ca^2+^ trigger apoptosis due to disruptions in complex regulatory pathways [28]. Further studies are necessary to confirm these findings and guide clinical practice.

Changes in cytosolic Ca^2+^ concentration via activation of some transient receptor potential (TRP) Ca^2+^ channels regulate cancer cell survival, apoptosis and metastasis [29]. Overexpression of TRPV1 channels in some breast cancer cell lines may contribute to breast cancer cell processes important in tumor progression such as angiogenesis [14]. Chelation of cytosolic Ca^2+^ dramatically reduces survival in breast cancer cells when compared to normal breast cells. This suggests that a high baseline cytosolic Ca^2+^ concentration is needed to support breast cancer cell survival and growth, although excessive activation of TRPV1 by exogenous agonists leads to cancer cell apoptosis [30]. Blocking Ca^2+^ influx through the TRPV1 ion channel results in a similar degree of decline in cell survival of all three cell lines in vitro. Concomitant use of sevoflurane, but not propofol, partially reverses this effect of TRPV1 antagonism. These findings suggest that changes in intracellular Ca^2+^ homeostasis play an important role in the general anesthetic-mediated enhancement of breast cancer cell survival. Specifically, the TRPV1 channel is a potential site of action of sevoflurane in altering intracellular Ca^2+^ levels leading to increased survival of breast cancer cells. Additional studies are needed to further elucidate the mechanism of the observed effect. While such in vitro studies help us elucidate the mechanisms that underlie anesthetic effects on breast cancer cell function, it is important to evaluate these relationships with animal studies and prospective randomized control trials.

In summary, exposure to sevoflurane, but not propofol, at clinically relevant concentrations and durations increased the survival of breast cancer cells in vitro. Breast cancer cells require higher cytoplasmic Ca^2+^ levels to maintain survival than normal breast tissue. Sevoflurane partially reverses the decrease in survival caused by TRPV1 antagonism suggesting that sevoflurane may enhance breast cancer cell survival through the activation of TRPV1 Ca^2+^ channels.

## Conclusion

Sevoflurane, but not propofol, at clinically relevant concentrations and durations increased the survival of breast cancer cells partially through the activation of TRPV1 Ca^2+^ channels. Breast cancer cells require higher cytoplasmic Ca^2+^ concentrations to maintain survival than normal breast tissue.

## Supplementary information

**Additional file 1.**

## Data Availability

The datasets used and/or analyzed during the current study are available from the corresponding author on reasonable request

## Abbreviations

TRPV1: Transient receptor protein V1
MTT: 3-(4,5-dimethylthiazol-2yl)-2,5-diphenyltetrazolium bromide
DMSO: dimethyl sulfoxide
BrdU: 5-bromo-2’deoxyuridine
