# Supplementary figures and images for: Sevoflurane modulates breast cancer cell survival via modulation of intracellular calcium homeostasis
Source: BMC Anesthesiol. 2020 Sep 29;20:253. doi: 10.1186/s12871-020-01139-y (PMC7526115; doi:10.1186/s12871-020-01139-y)

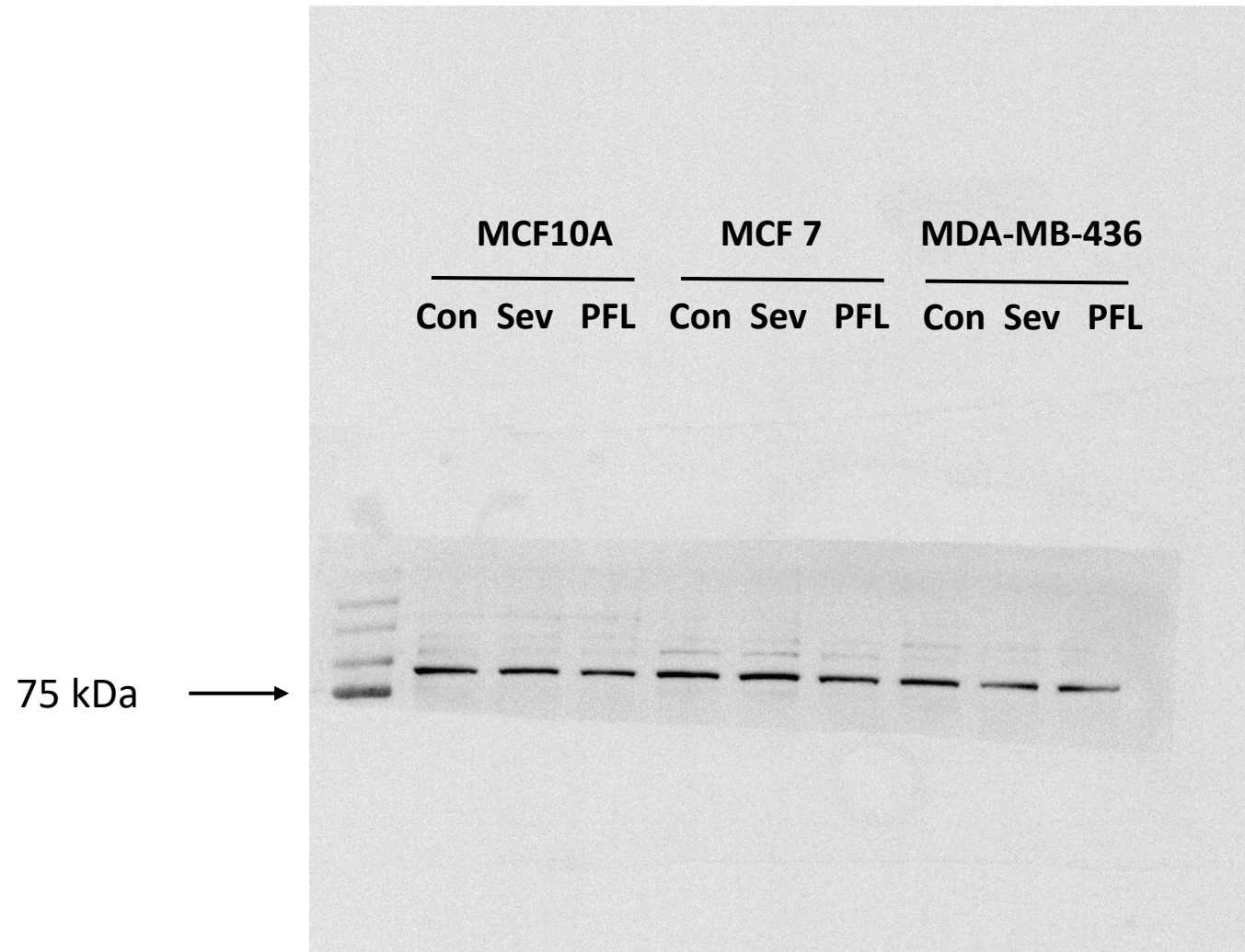

Blot #1: TRPV 1

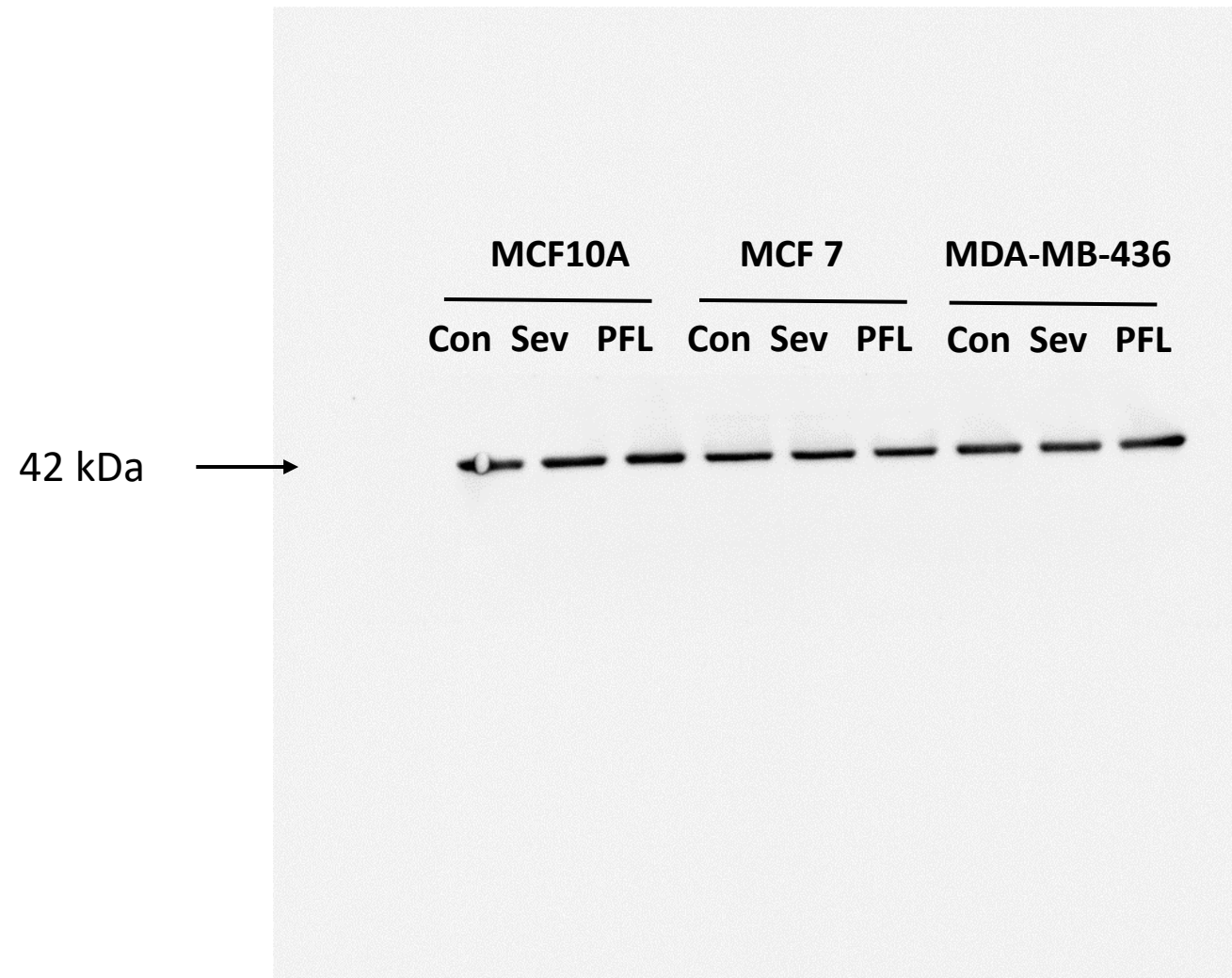

Blot #2:  $\beta$ -actin

Fig.8

Supplement: Supplementary file 1 — Additional file 1. [file 12871_2020_1139_MOESM1_ESM.pdf]
